# 

**DOI:** 10.1192/bjb.2023.2

**Published:** 2024-02

**Authors:** Katerina Denediou Derrer

**Affiliations:** is ST6 in general adult psychiatry at Hertfordshire Partnership Foundation Trust, Hatfield, UK. Email: katerinadenediou@yahoo.com

**Figure:**
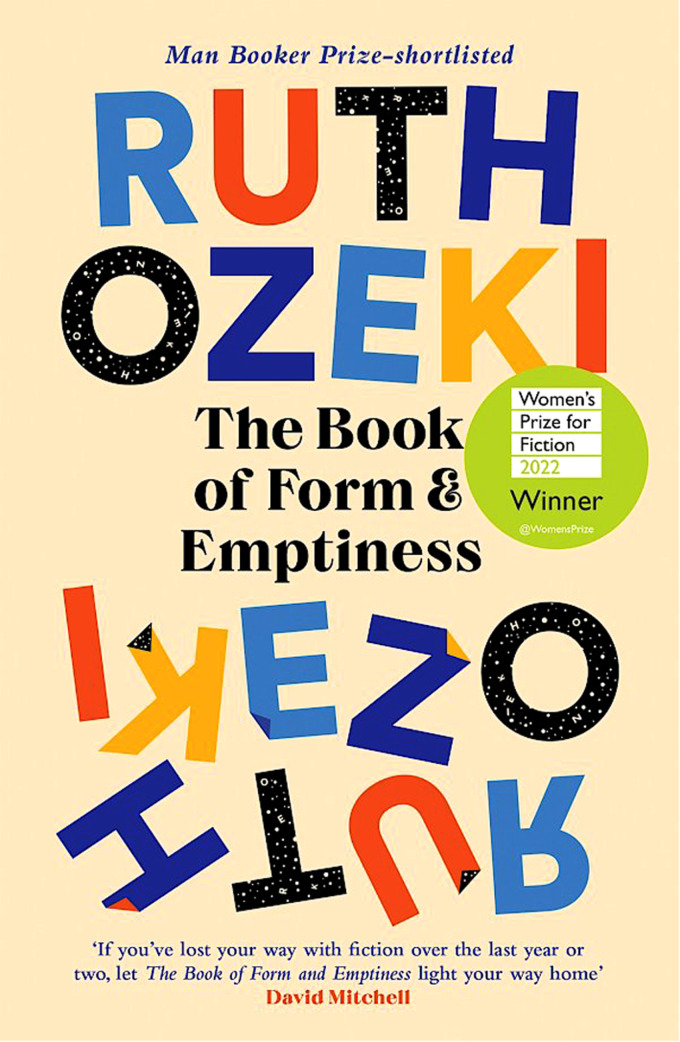

In this novel, Ozeki explores the nature of complicated bereavement. It starts with Benny and his mother suffering an unprocessed grief reaction following the tragic death of their beloved musician father and Japanese husband Kenji.

Teenager Benny is the main character in this book, which is set in an unnamed Western city. Benny's mother Annabelle welcomes signs and coincidences as messages communicated by Kenji, while she tries to hold on to anything that could keep his memory alive. She develops a web of overvalued ideas and becomes enclosed in her own reality of trying to survive financially in a world without job security, in a health system that withholds care from the uninsured or those who can't afford it.

Benny initially hears his father's voice at Kenji's cremation ceremony; he then starts hearing other noises and voices, almost exclusively of objects such as scissors and Christmas tree ornaments. In a maths class, despite trying to ignore what he hears, he sees a number swishing in front of his eyes. He hears the glass pane of the window apologise for being put there and unintentionally causing a bird to die on impact. The voice coming from the scissors becomes too intense and tells him to hurt his teacher. Benny retains reality testing, but when he decides to write his story he feels overwhelmed and suffers an accident that alerts his mother to the fact that he had been skipping school.

In this book we also meet a young woman with possible emotionally unstable personality disorder (EUPD) and an older homeless disabled poet who was previously diagnosed with schizophrenia. Benny identifies with the poet but is fearful of ending up like him.

I wonder why the author has chosen to portray the doctor unsympathetically. The doctor diagnoses the 14-year-old with attention-deficit hyperactivity disorder ADHD even though he has not exhibited any of the relevant symptoms. Ozeki also has the doctor dismissing the patient's reality, thereby refusing to believe how severe the psychosis was.

Ruth Ozeki compares the cultural processes of bereavement in the Western and Japanese cultures. What we could diagnose as complex bereavement or grief is interpreted differently according to different spiritual/religious or psychological frameworks.

